# *Maeropsis paphavasitae* and *Rotomelita longipropoda*, two new species (Crustacea, Amphipoda) from Lower Gulf of Thailand

**DOI:** 10.3897/zookeys.307.5273

**Published:** 2013-06-05

**Authors:** Koraon Wongkamhaeng, Charles Oliver Coleman, Pornsilp Pholpunthin

**Affiliations:** 1 Excellence Center for Biodiversity of Peninsular Thailand, Faculty of Science, Prince of Songkla University, 90112 Thailand; 2 Museum für Naturkunde Berlin, Invalidenstraße 43, 10115 Berlin, Germany; 3 Marine and Coastal Resources Institute (MACORIN), Prince of Songkla University, 90112 Thailand

**Keywords:** Crustacea, Amphipoda, Maeridae, Melitidae, *Maeropsis paphavasitae*, *Rotomelita longipropoda*, seagrass bed, Gulf of Thailand, new species, taxonomy

## Abstract

Two new species of maerid and melitid Amphipoda, *Maeropsis paphavasitae* and *Rotomelita longipropoda*, respectively, collected from a seagrassbed of the Lower Gulf of Thailand, are described. *Maeropsis paphavasitae* is characterized by it seven teeth on the palm of gnathopod 2 and *Rotomelita longipropoda* can be recognized by its long gnathopod 1 propodus. Their characters are described and illustrated. All specimens are deposited at Princess Maha Chakri Sirindhorn Natural History Museum, Prince of Songkla University, Thailand and the Museum für Naturkunde, Berlin.

## Introduction

The Gulf of Thailand contains many seagrass beds on the coast both the mainland and the islands along the gulf. There are 12 species of seagrass reported from this area. The seagrass habitats in Thailand have been investigated since 1902, covering various topics from both fauna and flora (Prathep et al. 2010). However, only one contribution deals with gammaridean amphipods, *Cheiriphotis trifurcata* was report (Wongkamhaeng et al. 2012). In this study, we describe the two new gammaridean species *Maeropsis paphavasita* sp. n. and *Rotomelita longipropoda* sp. n., both of which were found in the seagrass. The discovery of them represents the first record of these two genera in South China Sea. Figures and descriptions of both species are provided.

## Material and methods

**Figure 1.**
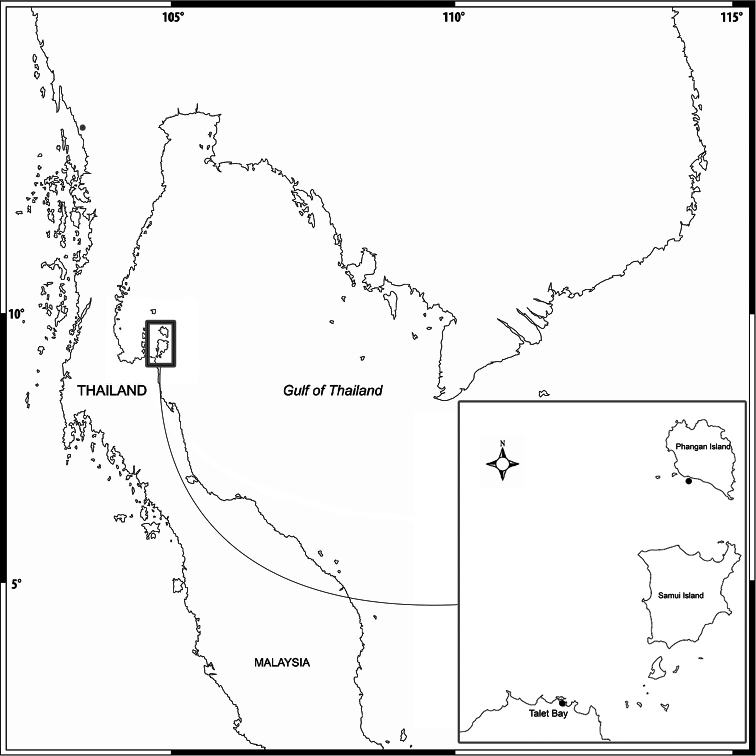
Map of sampling area.

Amphipods were collected using a 20×20cm^2^ Ekman’s grab in the mix of species seagrass beds of Talet Bay and Phangan Island (Figure 1). The sites were visited at low tide and the specimens were collected from the subtidal zone (2–5 m). The sediment was sieved with a 0.5 mm sieve. Amphipod specimens were sorted out and preserved in 70% ethanol. In the laboratory, the animals were examined using a compound microscope and later selected for dissection. The appendages were examined and ﬁgures were drawn using an Olympus CH30 light microscope with a camera lucida. The following abbreviations are used: A, antenna; G, gnathopod; HD, head; LL, lowerlip; MD, mandible; MX, maxilla; MP, maxilliped; P, pereopod; Pl, pleopod; T, telson; U, uropod; UR, urosome; UL, upperlip; r, right; l, left; ♂, male; ♀, female. The type material of the new species is deposited at Prince of Songkla University Zoological Collection (PSUZC) and the Museum für Naturkunde, Berlin (ZMB).

## Results

### Systematics Maeridae Krapp-Schickel, 2008

#### Maeropsis

Chevreux, 1919

http://species-id.net/wiki/Maeropsis

##### Diagnosis

**(modified from Krapp-Schickel 2008)**. Eyes reniform or round. Body smooth. Mandible palp article 1 with tooth-shaped distal prolongation, article 3 slender, linear. Maxilla 2 inner plate setation also present laterally. Gnathopods subchelate, both with well-defined palmar corner. Gnathopod 2 dactylus outer margin smooth; palm with small U-shaped excavation, palmar corner with tooth-shaped elevation and bearing. Pereopod dactyli simple. Uropod 3 with subequal rami, distally truncated, no second article on outer ramus visible. Epimera 1–3 smooth, with posterodistal tip. Telson cleft, lobes distally incised, outer end of incision clearly longer than inner one; with 1-2 setae inserted in incision.

##### Type species.

*Maeropsis perrieri* Chevreux, 1919 (type by monotypy).

##### Species composition.

*Maeropsis brevispina* (Kim & Kim, 1991); *Maeropsis cobia* Krapp-Schickel, 2009; *Maeropsis griffini* (Berents, 1983), *Maeropsis perrieri* Chevreux, 1919 (type species); *Maeropsis paphavasitae* sp. n. (this study); *Maeropsis rathbunae* (Pearse, 1908); *Maeropsis revelata* (Krapp et al., 1996); *Maeropsis serratipalma* (Nagata, 1965).

#### Maeropsis paphavasitae sp. n.

urn:lsid:zoobank.org:act:103B72E3-73B0-4DA4-A1CC-CC0BFD3D0C72

http://species-id.net/wiki/Maeropsis_paphavasitae

##### Type material.

*Holotype*. ♂, THAILAND, Lower Gulf of Thailand, Talet Bay (09°18'39.5"N, 99°46'46.4"E), seagrass bed (associated with *Thalassia hemprichii*), 1 May 2008, Puttapreecha, R., PSUZC-CR-0198.

*Paratypes*, collected with holotype ZMB 27979 (3♀); PSUZC-CR-0199 (3♂; 5♀)

##### Description.

Based on male holotype. Body length 5.1 mm (from tip of rostrum to apex of telson). *Body* compressed, subcylindrical. *Head*, lateral cephalic lobe truncate, without rostrum, eyes round. *Antenna 1*, ratios of peduncular articles 1–3 9:10:2.5; peduncular article 1 with a ventromarginal robust seta and a distoventral robust seta; accessory flagellum with 6 articles.

*Upper lip*, (labrum) distally rounded. *Lower lip*, inner lobes small, pubescent; outer lobes covered with thin hair-like setae. *Mandible*, both incisors with 7 teeth; lacinia mobilis armed with 5 teeth on the left side and 4 teeth on the right side; molar short, cylindrical; palp 3-articulate with ratios of 4:9:3, article 2 with marginal setae and article 3 with apical setae. *Maxilla 1*, inner plate with 3 plumose setae apically and marginal fine setae medially; outer plate with 6 serrate robust setae apically; palp 2-articulate with apical fine setae. *Maxilla 2*, inner plate with lateral and medial marginal setae; outer plate broader than inner plate, distally setose. *Maxilliped*, inner plate with 9 plumose setae; outer plate semi-oval, inner margin with 9 robust setae distally; palp 4-articulate, article 3 with 15 fine setae, article 4 with 6 fine marginal setae.

**Pereon.** *Gnathopod 1* subchelate, smaller than gnathopod 2; coxa anterodistally produced; basis posterior margin with 3 setae, longer than ischium and merus combined; carpus posterior margin and lateral surface densely setose; palm oblique. *Gnathopod 2*, basis with 2 posterior marginal setae; ischium and merus short; carpus triangular; propodus robust, subrectangular, 1.5 × longer than broad with posterior marginal plumose setae, palm transverse with 6 blunt teeth and an acute palmar corner, inner face bearing 1 subposterodistal robust seta; dactylus fits with palm, inner margin smooth, outer margin with 1 long seta. *Pereopod 3–4* similar to each other, basis with anterior and posterior marginal setae. *Pereopod 5* basis oval, posterior margin slightly produced distally, posterodistal lobe rounded. *Pereopod 6–* 7 similar, basis broader than pereopod 5, posterodistally produced with short anterior and posterior marginal setae; dactyli curved.

**Pleon.** *Epimera 1–2* posteroventral corner smooth, not produced. *Epimeron 3* produced posteroventrally, with 3 short ventral setae. *Uropod 1* longest, peducle longer than rami, posterior margin fringed with robust setae, anterior with 1 basofacial robust seta. *Uropod 2*, peduncle shorter than both rami; inner ramus longer than outer ramus. *Uropod 3*, peduncle shorter than rami; inner ramus subequal to outer ramus, distally truncate; inner ramus bearing only 1 proximal robust seta and 4 terminal setae. Telson longer than wide, cleft more than 95% of its length, lobes distally incised, outer end of incision longer than inner one, with 3 long apical setae and 2 lateral plumose setae.

**Female.** (sexually dimorphic characters). No sexual difference.

**Figure 2.**
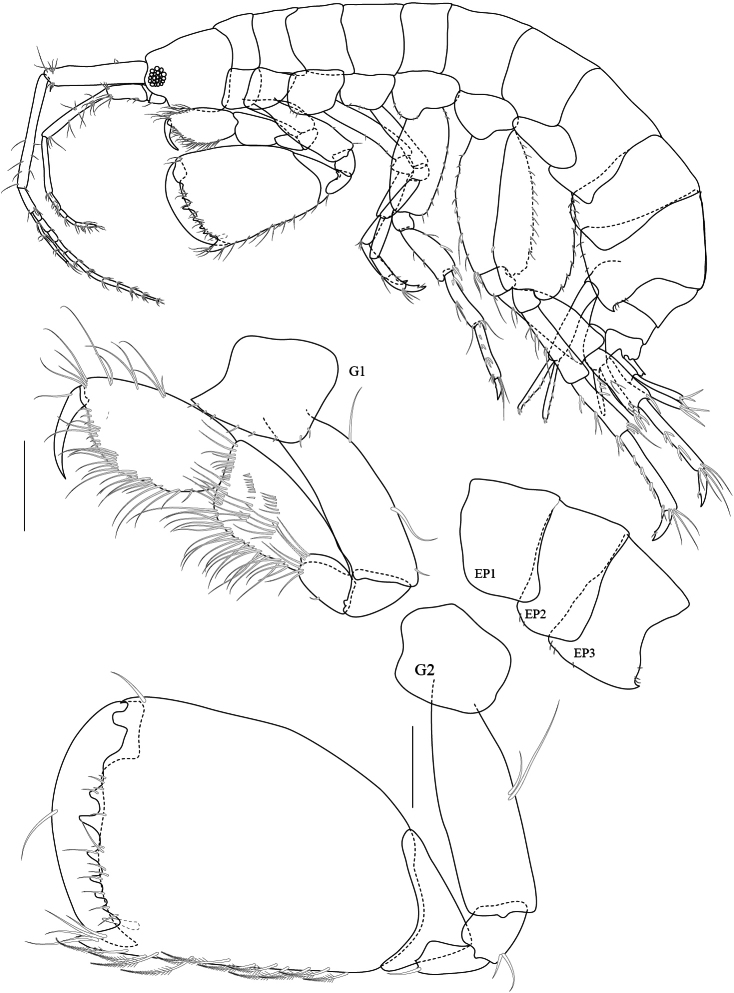
*Maeropsis paphavasitae* sp. n. holotype, male, (PSUZC-CR-00198), 5.1 mm. Talet Bay, Lower Gulf of Thailand. All scales represent 0.2 mm.

**Figure 3.**
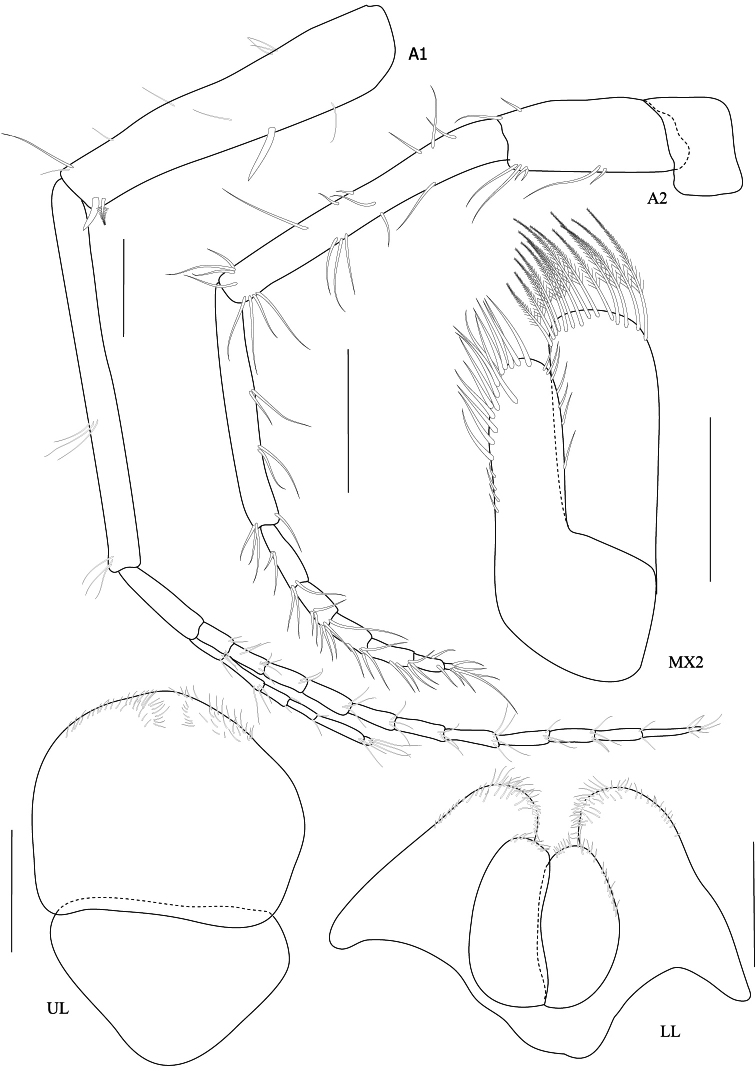
*Maeropsis paphavasitae* sp. n. holotype, male, (PSUZC-CR-00198), 5.1 mm. Talet Bay, Lower Gulf of Thailand. Scales for A1 and A2 represent 0.2 mm; MX2, LL, UL represent 0.1 mm.

**Figure 4.**
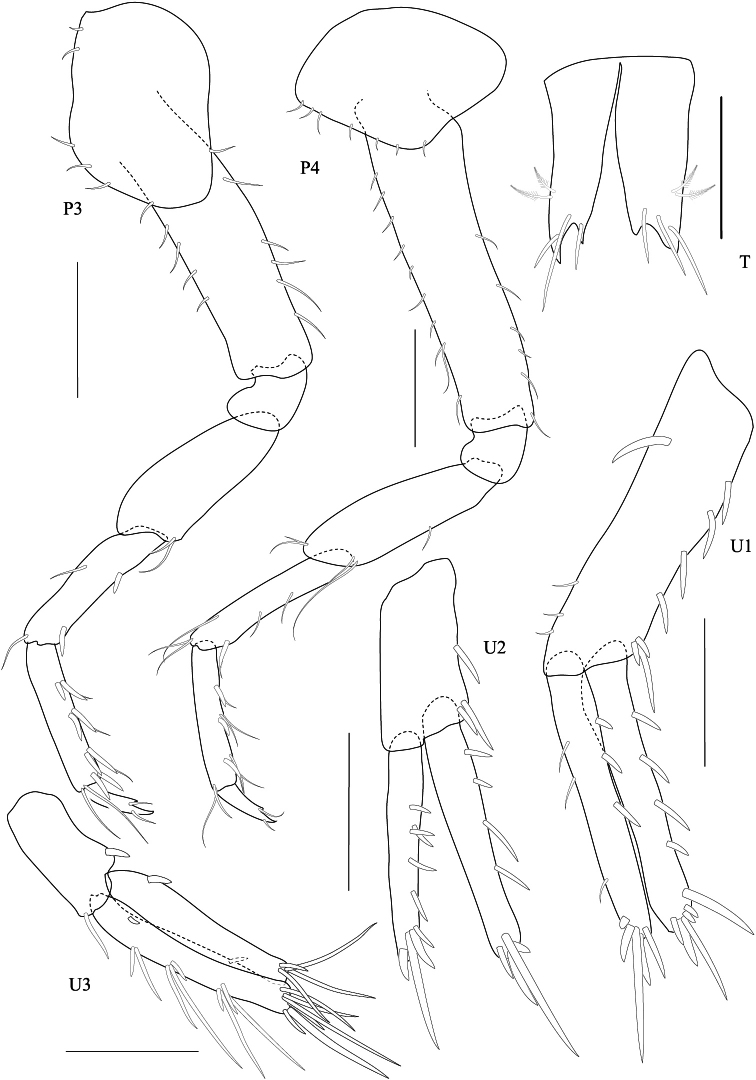
*Maeropsis paphavasitae* sp. n. holotype, male, (PSUZC-CR-00198), 5.1 mm. Talet Bay, Lower Gulf of Thailand. Scales for P3, P4, U1-3 represent 0.2 mm; T represents 0.1 mm.

**Figure 5.**
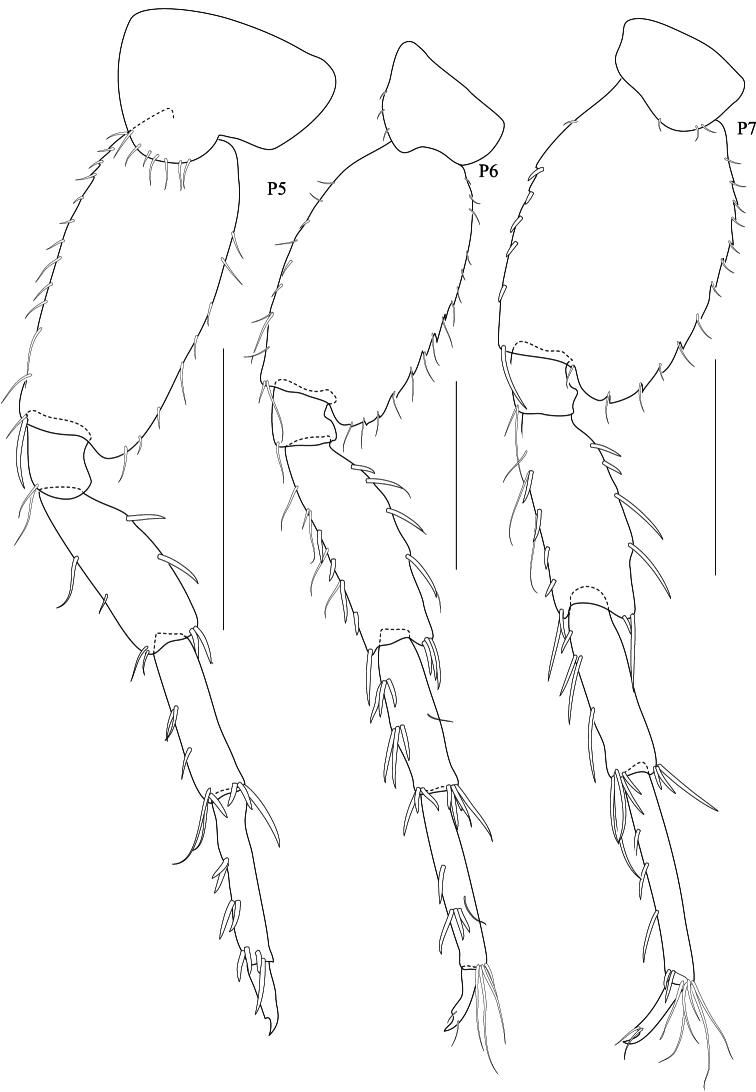
*Maeropsis paphavasita* sp. n. holotype, male, (PSUZC-CR-00198), 5.1 mm. Talet Bay, Lower Gulf of Thailand. All scales represent 0.5 mm.

**Figure 6.**
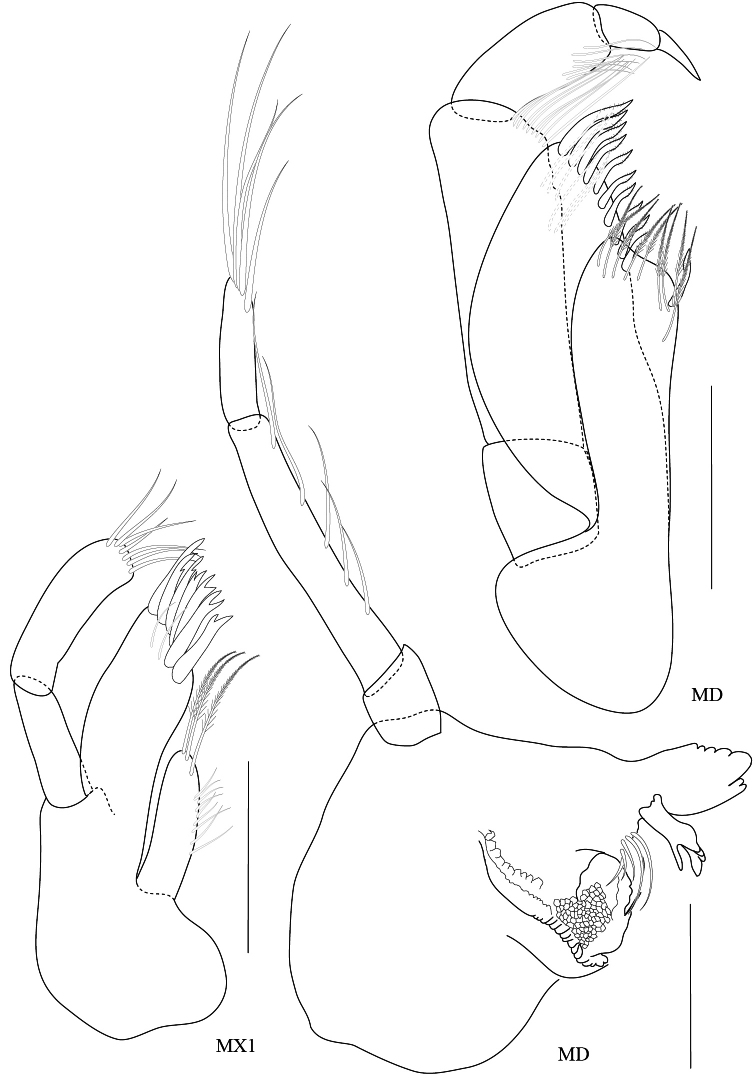
*Maeropsis paphavasita* sp. n. holotype, male, (PSUZC-CR-00198), 5.1 mm. Talet Bay, Lower Gulf of Thailand. All scales represent 0.1 mm.

##### Etymology.

The species is named in honor of Associate Professor Nittharatana Paphavasit of Chulalongkorn University, Thailand who contributed to the knowledge on seagrass habitats in Thailand.

##### Remarks.

*Maeropsis paphavasitae* sp. n. is very similar to *Maeropsis serratipalma* (Nagata, 1965) in the palm of gnathopod 2, which is transverse with 6 blunt teeth and a large defining tooth in both species. But the new species can be distinguished from *Maeropsis serratipalma* by the following characters: gnathopod 1 palm without clear defining palmar corner; gnathopod 2, merus not produced into a posterodistal tooth (vs. produced); propodus of gnathopod 2 subtriangular (vs. subrectangular), palm inner surface with 1 subposterodistal robust seta (vs. without); dactyli of pereopods 3-7 curved and smooth (vs. bearing two minute teeth); uropod 3 inner ramus with only 1 proximal seta (vs. armed with several marginal robust setae) and telson longer than broad (vs. broader than long).

### Melitidae Bousfield, 1973

#### Rotomelita

J.L. Barnard, 1977

http://species-id.net/wiki/Rotomelita

##### Diagnosis

**(from Barnard 1977).** Accessory flagellum 3+ articulate; mandibular palp thin, weak, articles linear, article 3 bearing few apical setae only; lower lip with small but fully discrete inner lobes; inner plate of maxilla 1 with only terminal setae; inner plate of maxilla 2 lacking medial setae; maxillipedal palp 4-articulate, article 4 unguiform; gnathopods ordinary, gnathopod 2 larger than gnathopod 1, lacking fuzz on article 5; uropod 3 greatly overreaching uropod 1, inner ramus small and scale-like, outer ramus immensely elongate, bearing short article 2; telson short, cleft, lobes very broad and apically truncate; urosomite 1 bearing 1 subdorsal spine on each side, otherwise pleonites dorsally smooth; anterior coxae (1–4) longer than posterior coxae (5–7); some gills pediculate.

##### Type species.

*Rotomelita lokoa* J.L. Barnard, 1977 (type by original designation).

##### Species composition.

*Rotomelita ana* J.L. Barnard, 1977; *Rotomelita lokoa* J.L. Barnard, 1977; *Rotomelita longipropoda* sp. n. (this study).

#### Rotomelita longipropoda sp. n.

urn:lsid:zoobank.org:act:DAC77E5F-B08A-4640-96B9-A269E74ADABB

http://species-id.net/wiki/Rotomelita_longipropoda

##### Type material.

*Holotype*. ♂ (1.65 mm), THAILAND, Lower Gulf of Thailand, Phangan Island (9°41'48"N, 100°0'2"E), seagrass bed (associated with *Thalassia hemprichii* and *Halophila ovalis*), 1 July 2009, Bantiwiwatkul, N., PSUZC-CR-0195.

*Allotype*. ♀, collected with holotype, PSUZC-CR-0196 (gravid female, 1.66 mm) *Other material*. Same data as for holotype, PSUZC-CR-0197 (5♂; 5♀)

##### Description.

Based on male holotype. Total body length 1.65 mm (from tip of rostrum to apex of telson). *Body*, rather slender and subcylindrical. *Head*, slightly shorter than first 2 pereonites; rostrum not developed; inferior antennal sinus shorter than eyes, concave, about 0.2 times head length; eye distinct. *Antenna 1*, longer than antenna 2, ratios of peduncular articles 1–3 2:2:1; article 1 slender; flagellum with 16 articles plus 1 rudimentary article, 2 times as long as peduncle; accessory flagellum with 3 articles, last article scale-like. *Antenna 2*, peduncle slender; gland cone fleshy, short, not reaching to end of peduncular article 3; articles 2-5 ratios 1:1:3:3; inner margin of article 4 and 5 with sparse setae; article 5 slightly shorter than 4; flagellum short with sparse setae, longer than peduncular article 5, composed of 7 articles, last article scale-like.

*Upper lip* (labrum), round and broad. *Lower lip*, inner lobe small, outer lobe pubescent, mandibular process well developed. *Mandible*, left incisor with 6 teeth and right incisor with 5 teeth; lacinia mobilis armed with 4 teeth on both sides; molar triturative; palp 3-articulate with ratios 1:2:3, article 3 with 3 apical setae. *Maxilla 1*, inner plate triangular, small, with 3 apical robust setae, outer plate with 8 apical serrate robust setae; palp 2-articulate, article 1 shorter than 2, article 2 apical margin with 4 robust setae. *Maxilla 2*, inner plate with 5 slender apical setae; outer plate subequal to inner plate, with 8 slender setae. *Maxilliped*, inner plate narrow, short, reaching half of outer plate, apically provided with 6 plumose setae and 3 stout robust setae; outer plate broad, subrectangular, almost reaching palp article 3, with 5 apical and marginal setae and 2 apical robust setae; palp 4-articulate, with ratios 4.5:4:4:3, article 4 unguiform.

**Pereon**. *Gnathopod 1*, subchelate, smaller than gnathopod 2; coxal plate subrectangular; length ratios of articles from basis to dactylus about 10:4:4:7:6:4; basis slender, posterior margin bearing long setae; ischium short, subrectangular with short posteromarginal setae; merus subrectangular with long and short posteromarginal setae; carpus trapezoidal with long setae on posterior margin; propodus subtriangular, shorter than carpus, palm transverse, produced, with sparse setae, posterodistal corner minutely serrate, slightly bending inwards; dactylus shorter than palm, falcate. *Gnathopod 2*, subchelate; coxal plate short and wide, subrectangular, length ratios of articles from basis to dactylus about 14:5:5:6:16:12; basis slender, 3 times as long as wide, anterior margin straight; ischium subrectangular, subequal to merus; carpus triangular; propodus enlarged, oval, 2 times as long as wide, palm strongly oblique with sparse setae; dactylus falcate, slightly shorter than palmar margin, inner margin smooth, the inner surface not excavate.

*Pereopod 3*, slender and elongate; coxal plate small and subtrapezoidal, with 5 fine setae on anterioroventral margin; length ratios of articles from basis to dactylus 10:2:6:5:6:2; basis slender; ischium short, subrectangular; merus slightly longer than carpus, produced anteriorly; carpus slender with apical setae; propodus subrectangular; basis to propodus bearing sparse setae on both sides; dactylus falcate, shorter than propodus. *Pereopod 4*, similar to pereopod 3, coxal plate subrectangular with fine setae on anteroventral margin; length ratios of articles from basis to dactylus about 12:2:6:5:6:2; basis slender; ischium short, subrectangular; merus produced anterodistally; carpus slender, subequal to propodus; basis to propodus with sparse setae on both margins; propodus long and narrow; dactylus falcate and short. *Pereopod 5*, shortest; coxa bilobed; length ratios of articles from basis to dactylus about 8:2:5:4:6:3; basis subrectangular with short fine setae on posterior margin; ischium shortest; merus with a posterodistal robust seta and 1 anterodistal seta; carpus with distal robust setae on both sides; propodus with 4 distal long fine setae; dactylus short and curved. *Pereopod* 6 elongate, 1.6 times as long as pereopod 5; coxa posteriorly produced with rounded lobe; length ratios of articles from basis to dactylus about 12:3:8:7:11:4; basisposterior margin straight with minute castellations, with fine setae on both margins; ischium short with fine setae on anteroventral corner; merus oblong, with robust setae on posterior margins and posterodistal corner; carpus subrectangular with 3 anterodistal robust setae; propodus oblong, slender with marginal robust setae, setose posterodistally; dactylus falcate. *Pereopod 7*, subequal to pereopod 6; coxa short and wide, semicircular; length ratios of articles from basis to dactylus about 12:3:8:8:14:5; basis posteriodistally produced, bearing fine setae on posterior margin; ischium short and subquadrate; merus elongate with robust setae on both sides; carpus subequal to merus, bearing 3 anterodistal robust setae; propodus oblong, longer than merus, bearing robust setae on both margins and anterodistal corner; dactylus falcate.

**Pleon**. *Epimera 1–3* each with small posterodistal tooth. *Uropod 1*, peduncle with 2 distal robust setae; rami slightly shorter than peduncle, armed with 3 apical robust setae. *Uropod 2*, peduncle subequal to rami; rami subequal, outer ramus with a marginal robust seta and apex armed with several long and short robust setae. *Uropod 3*, biramous; inner ramus minute, pointed apically, bearing 1 apicomedial robust seta; outer ramus biarticulate, with marginal robust setae, distal article short. *Telson*, broader than long, cleft, lobes very broad and apically truncate, each lobe with 2 apical setae.

**Female**. (allotype) (sexually dimorphic characters). Total body length 1.66 mm (from tip of rostrum to apex of telson).

**Pereon.** *Gnathopod 1*, subchelate, smaller than gnathopod 2; coxal plate subrectangular; length ratios from basis to dactylus about 10:4:4:6:5:4; propodus subtriangular, longer than dactylus, palm transverse with short marginal setae; dactylus falcate, tapering distally. *Gnathopod 2*, subchelate; length ratios from basis to dactylus about 10:5:5:6:8:5; basis slender, 2.5 times as long as wide, anterior margin weakly produced with sparse setae; ischium subrectangular; carpus triangular; propodus suboval, 1.6 times as long as wide, palm oblique, defined by 2 robust setae; dactylus falcate, slightly shorter than palmar margin, inner margin smooth.

**Figure 7.**
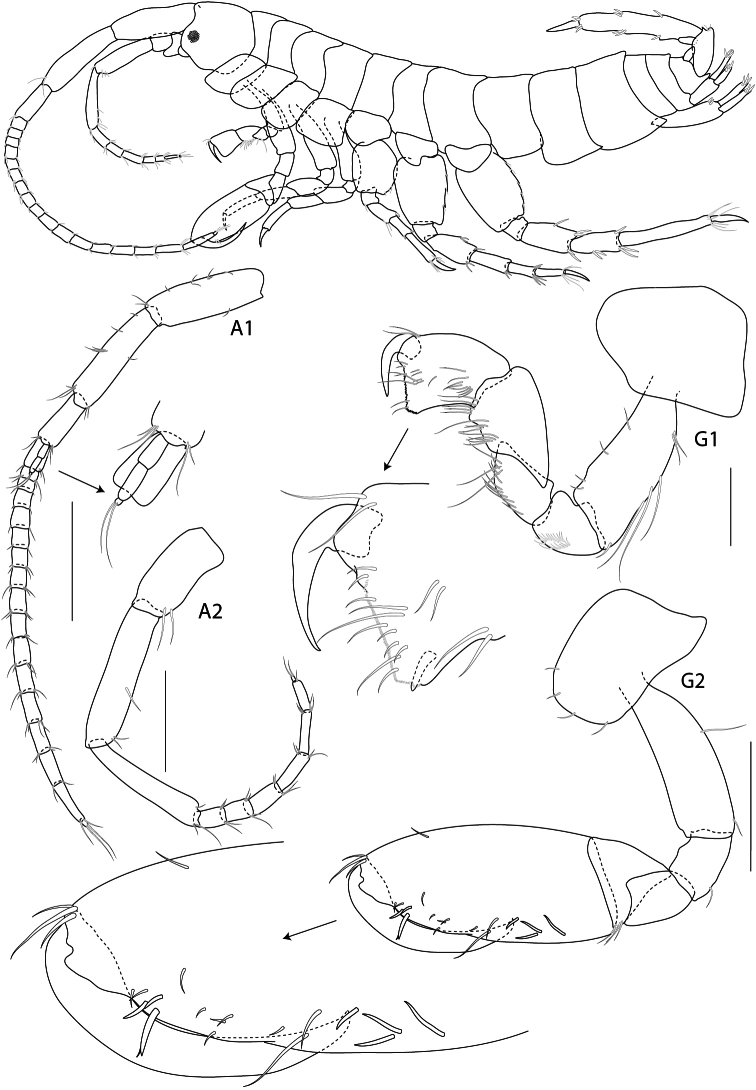
*Rotomelita longipropoda* sp. n. holotype, male, (PSUZC-CR-00195), 1.65 mm, Phangan Island, Lower Gulf of Thailand. All scales represent 0.2 mm.

**Figure 8.**
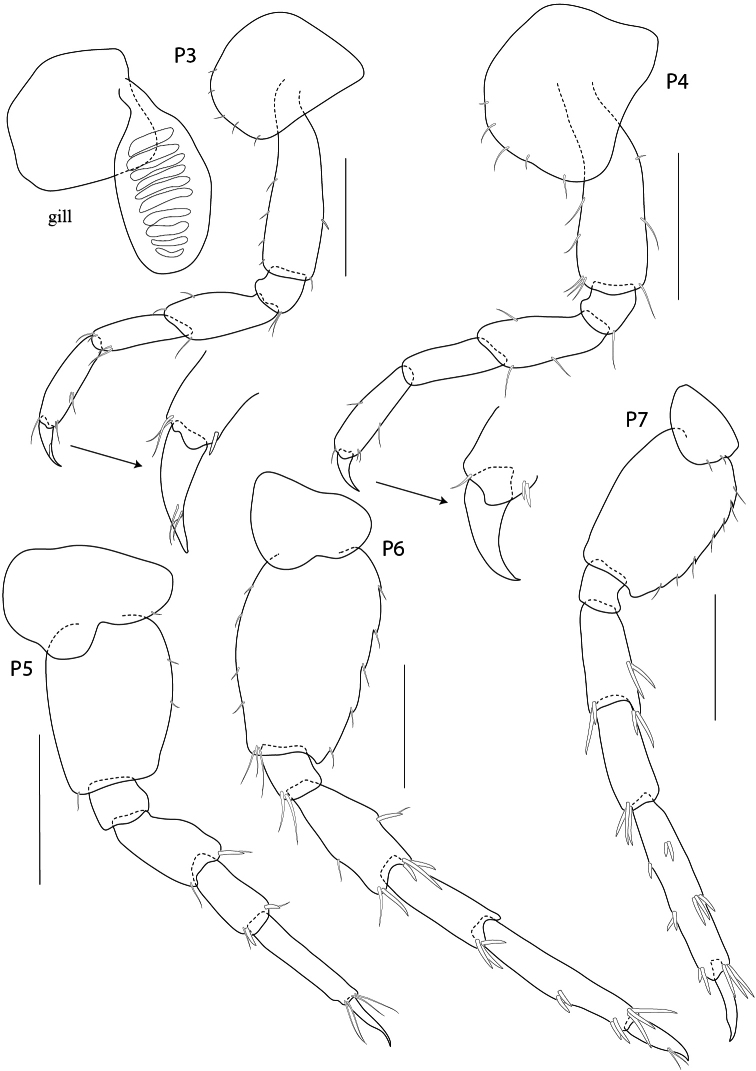
*Rotomelita longipropoda* sp. n. holotype, male, (PSUZC-CR-00195), 1.65 mm, Phangan Island, Lower Gulf of Thailand. All scales represent 0.2 mm.

**Figure 9.**
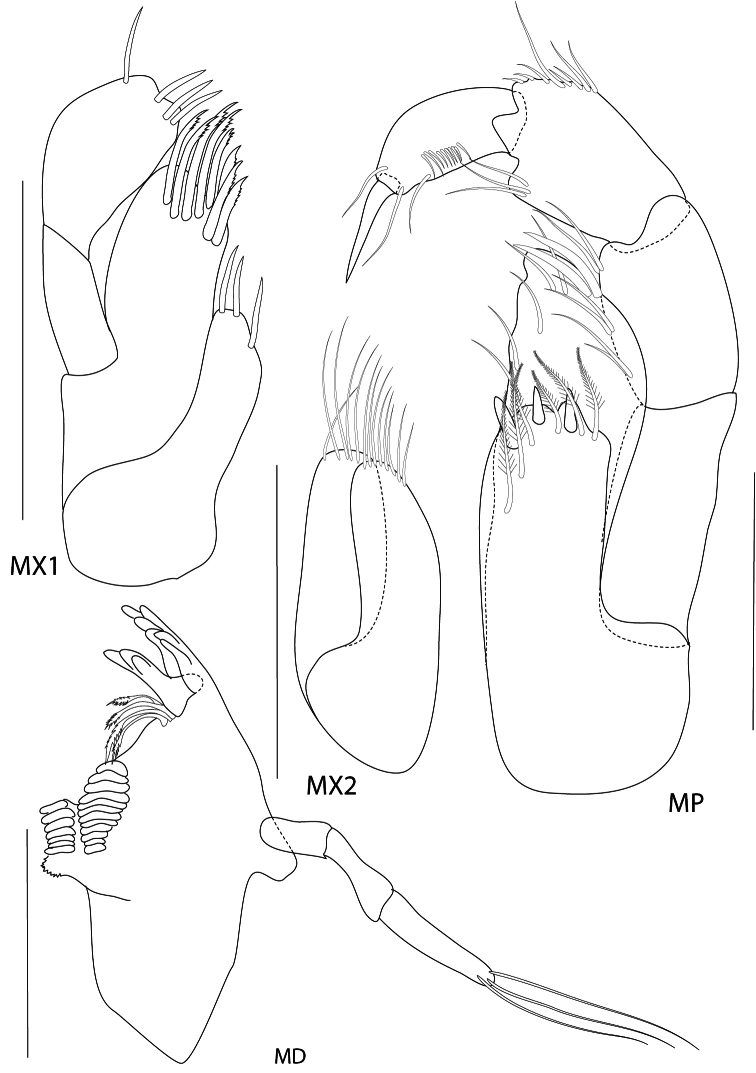
*Rotomelita longipropoda* sp. n. holotype, male, (PSUZC-CR-00195), 1.65 mm, Phangan Island, Lower Gulf of Thailand. All scales represent 0.1 mm.

**Figure 10.**
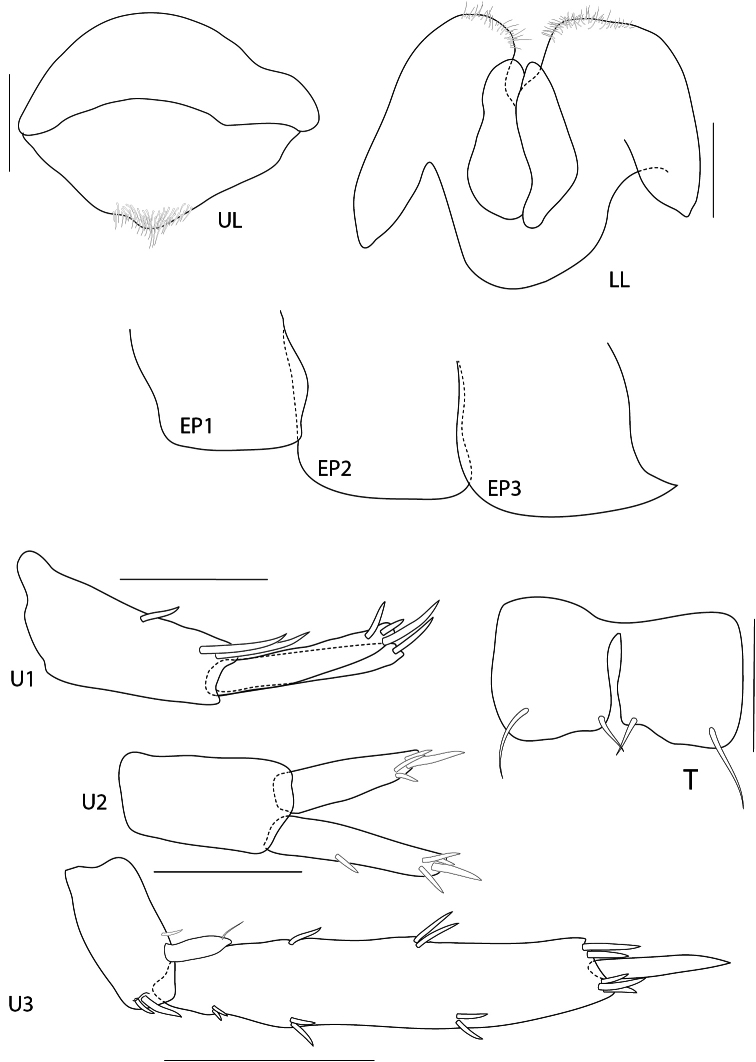
*Rotomelita longipropoda* sp. n. holotype, male, (PSUZC-CR-00195), 1.65 mm, Phangan Island, Lower Gulf of Thailand. All scales represent 0.05 mm.

**Figure 11.**
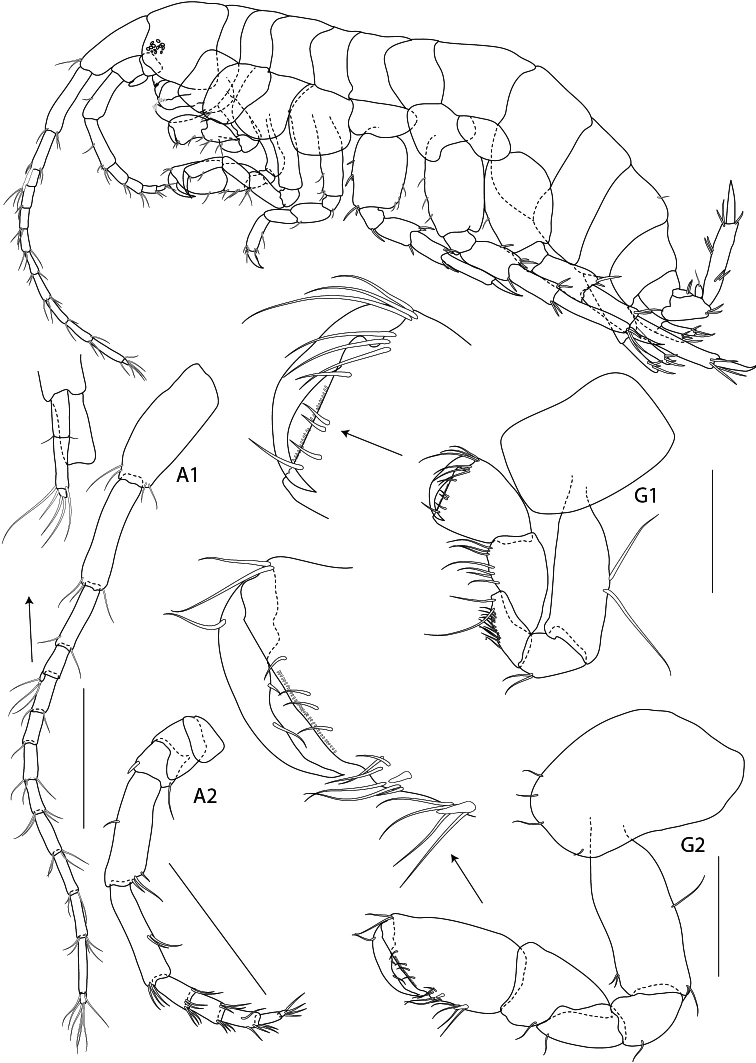
*Rotomelita longipropoda* sp. n. allotype, female, (PSUZC-CR-00196), 1.66 mm, Phangan Island, Lower Gulf of Thailand. All scales represent 0.2 mm.

##### Etymology.

The specific name “longipropoda” is from latin ‘longi = long’ and ‘propoda = propodus’, referring to the relatively long propodus of male gnathopod 2 compared to congeners.

##### Remarks.

*Rotomelita longipropoda* new species shares some characteristics with *Rotomelita lokoa* Barnard, 1977 and *Rotomelita ana* Barnard, 1977 from Hawaii by having stalked coxal gills; weakly sexually dimorphic gnathopod 1 and 2; coxa 1 not expanded distally; coxa 4 proximally excavated, smooth pleon segments 1-3 and urosomite 1, and a deeply cleft telson, with truncate lobes.

However, the new species can be easily distinguished from its congeners by having eyes (vs. lacking eyes in *Rotomelita lokoa* and *Rotomelita ana*); antenna 1 peduncle without robust setae (vs. with 2 robust setae on ventroproximal margin in *Rotomelita lokoa* and *Rotomelita ana*); male gnathopod 2 with relatively long propodus (2.7 times as long as carpusvs, 1.6 times in the two other species) and the uropod 3 rami are shorter (2.2 times as long as peduncle) compared to the other two species (3.3 times as long as peduncle).

*Rotomelita longipropoda* is also similar to other *Rotomelita* subgroup i.e. *Nainaloa* Karaman & Barnard, 1979 and *Tegano* Barnard & Karaman, 1982. *Rotomelita longipropoda* can be distinguished from *Nainaloa* as follows: *Rotomelita longipropoda* gnathopod 1 palm has inner surface not excavate (inner face of propodus excavate in *Nainaloa*); pediculate gills (figure 8) (simple gill in *Nainaloa*); article 2 of pereopods 5—7 not lobed (lobed in *Nainaloa*) and truncate lateral cephalic lobes (prominent in *Nainaloa*). Besides, *Rotomelita longipropoda* is also allied to member of genus *Tegano* occurring in Indo-Pacific. They differ as follows: *Rotomelita longipropoda* has mandibular palp article 3 longer than article 2, bearing 3 apical setae (article 3 reduced with 1 apical seta in *Tegano*) and the telson short, cleft, lobes very broad and apically truncate (tapering and apically point in *Tegano*).

In terms of ecology *Rotomelita*, *Nainalao* and *Tegano* are recorded from fresh to brackish water (Barnard 1977; Bousfield 1971 and Lowry and Springthorpe 2009) while *Rotomelita longipropoda* was collected from seagrass beds in a salinity range of 28—32 ppt. However, there is a small creek located 1 km northern of seagrass patch. The amphipods also can be considered as a brackish species. It is the first record of this genus from West Pacific.

## Supplementary Material

XML Treatment for Maeropsis

XML Treatment for Maeropsis paphavasitae

XML Treatment for Rotomelita

XML Treatment for Rotomelita longipropoda
